# 922. Cost-effectiveness of baloxavir marboxil versus oseltamivir or no treatment for the management of influenza in the United States

**DOI:** 10.1093/ofid/ofad500.967

**Published:** 2023-11-27

**Authors:** Svenn Hansen, Shih-Chen Cheng, Andy Surinach, Vince Yau, Jennie H Best, Hassan Zaraket, Hao Zhou, Marie-Helene Blanchet Zumofen

**Affiliations:** F. Hoffmann-La Roche Ltd, Basel, Basel-Stadt, Switzerland; Genentech, South San Francisco, California; Genesis Research, Hoboken, New Jersey; Genentech, South San Francisco, California; Genentech, Inc., South San Francisco, California; Roche Products Ltd, Welwyn Garden City, England, United Kingdom; Genentech, South San Francisco, California; F. Hoffman La Roche, Basel, Basel-Stadt, Switzerland

## Abstract

**Background:**

Influenza poses a significant burden that can be effectively mitigated with antivirals. Baloxavir marboxil is an oral, single dose first-in-class endonuclease inhibitor that improves influenza symptoms and rapidly reduces virus shedding, shortening the infectious period and potentially reducing viral transmission. This study used real-world data to inform a cost-effectiveness (CE) model of antiviral treatment with baloxavir vs oseltamivir or no treatment.

**Methods:**

A decision tree CE model was developed for seasonal influenza between 2018 and 2020. Patients aged 12 and older could receive baloxavir, oseltamivir or no treatment (Figure 1). Outcomes included complications, recovery and death. Patient profile and characteristics, complications and costs were derived from the Merative™ MarketScan® Research Databases, including US commercial claims, Medicare and Medicaid Supplemental databases. Healthcare resource use included outpatient visits, hospitalizations and intensive care unit admissions.

Clinical inputs, recovery rates and utilities were derived from the literature. The base case model used a lifetime time horizon with 3.0% discounting for costs and 2.7% for quality-adjusted life-years (QALYs), assuming no transmission reduction. A subgroup analysis was conducted for otherwise healthy (OwH) and high-risk groups; scenario analyses explored the impact of reduced viral transmission with baloxavir.

**Results:**

In the base case analysis, baloxavir resulted in an incremental CE ratio (ICER) of $7788/QALY vs oseltamivir and $311/QALY vs no treatment; subgroup analyses showed even greater CE in the high-risk population (Table 1). Scenario analyses showed increasing net monetary benefit (NMB) with incremental reductions in viral transmission with baloxavir, where a 5% reduction in transmission yielded a NMB for baloxavir of $4153 vs oseltamivir and $13,098 vs no treatment (Figure 2).

**Figure:**
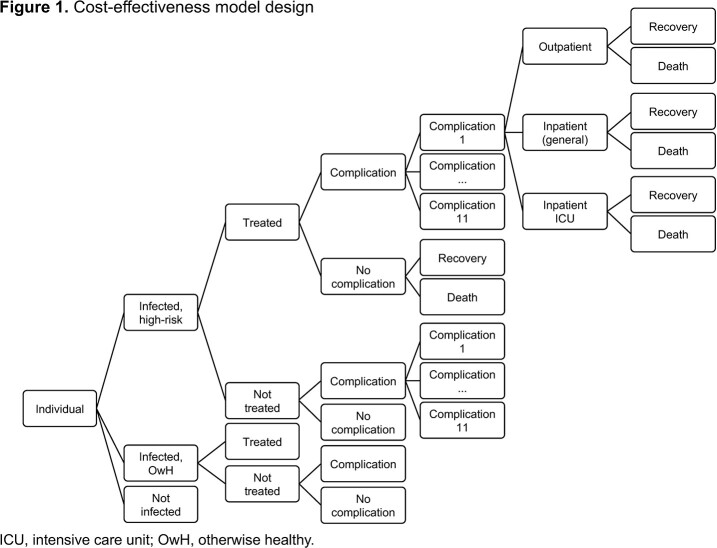

**Conclusion:**

This real-world evidence-driven model showed that baloxavir is cost-effective vs oseltamivir or no treatment from a US payer perspective. Transmission reductions with baloxavir can have a substantial health economic benefit, and baloxavir may play an important role in management of seasonal influenza and pandemic preparedness.

**Figure:**
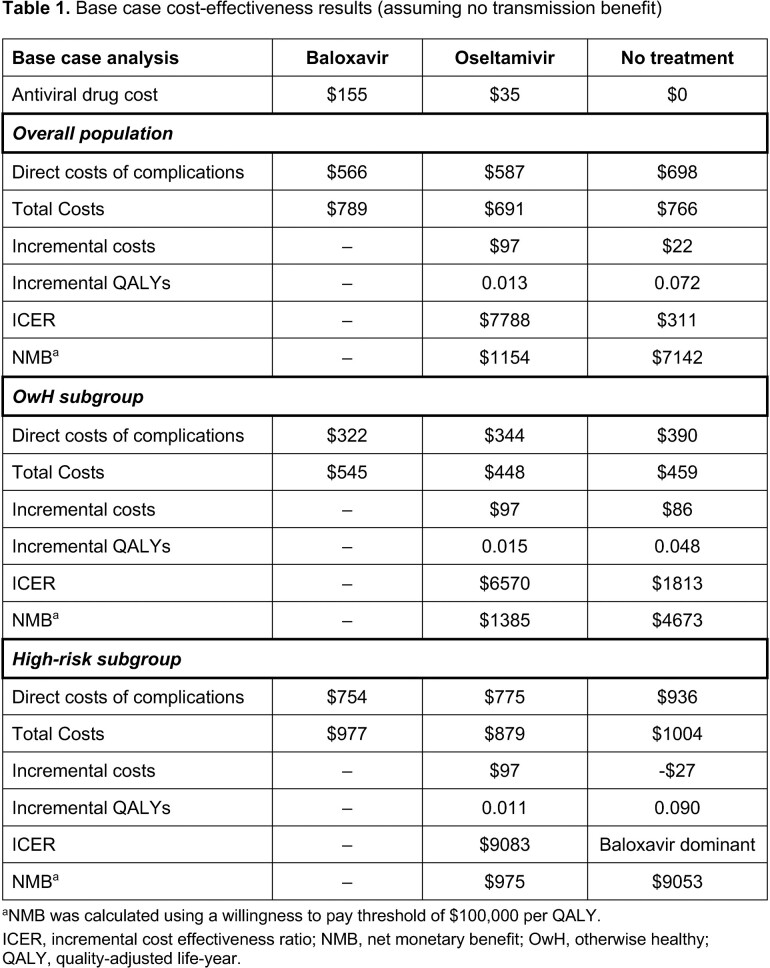

**Figure:**
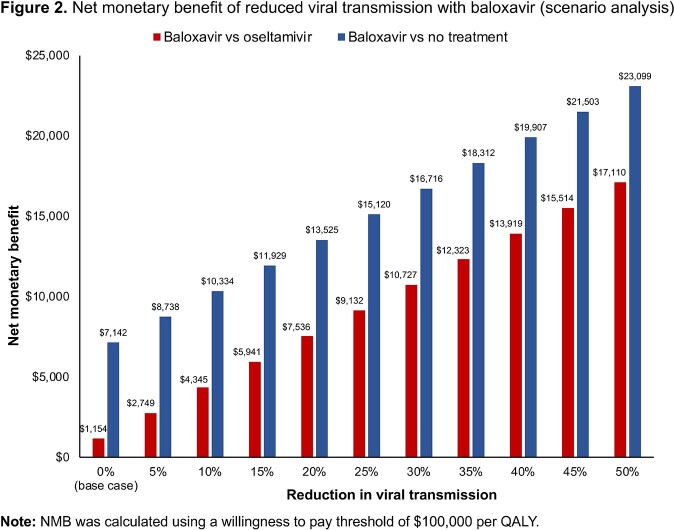

**Disclosures:**

**Svenn Hansen, n/a**, F. Hoffman La Roche: Employee|F. Hoffman La Roche: Stocks/Bonds **Shih-Chen Cheng, M.S., P.h.D**, Genentech/F. Hoffman La Roche: Genentech Employee|Genentech/F. Hoffman La Roche: Stocks/Bonds **Andy Surinach, MPH**, Genentech/Roche: employee of Genesis Research which receives funding from Genentech/Roche for consulting services **Vince Yau, PhD**, Genentech/F. Hoffman La Roche: Genentech Employee|Genentech/F. Hoffman La Roche: Stocks/Bonds **Jennie H. Best, PhD**, Genentech: Stocks/Bonds **Hassan Zaraket, PhD**, F. Hoffman La Roche: Employee|F. Hoffman La Roche: Stocks/Bonds **Hao Zhou, PhD**, Genentech/F. Hoffman La Roche: Genentech Employee|Genentech/F. Hoffman La Roche: Stocks/Bonds **Marie-Helene Blanchet Zumofen, PhD MBA**, F. Hoffman La Roche: Employee|F. Hoffman La Roche: Stocks/Bonds

